# Mycobiome analyses of critically ill COVID-19 patients

**DOI:** 10.1128/spectrum.04110-23

**Published:** 2024-12-19

**Authors:** Danielle Weaver, Sara Gago, Matteo Bassetti, Daniele Roberto Giacobbe, Juergen Prattes, Martin Hoenigl, Florian Reizine, Hélène Guegan, Jean-Pierre Gangneux, Michael John Bromley, Paul Bowyer

**Affiliations:** 1 University of Manchester, Manchester, United Kingdom; 2 Department of Health Sciences (DISSAL), University of Genoa, Genoa, Italy; 3 Infectious Diseases Unit, IRCCS San Martino Polyclinic Hospital, Genoa, Italy; 4 Division of Infectious Diseases, Medical University of Graz, Graz, Austria; 5 Biotech Med, Graz, Austria; 6 Translational Medical Mycology Research Unit, ECMM Excellence Center for Medical Mycology, Medical University of Graz, Graz, Austria; 7 Medical Intensive Care Unit, Centre Hospitalier Universitaire de Rennes, Rennes, France; 8 CHU Rennes, Inserm, EHESP, Institut de Recherche en Santé, Environnement et Travail (IRSET), Université de Rennes, Rennes, France; 9 Centre Hospitalier Universitaire de Rennes, Laboratoire de Parasitologie-Mycologie, Centre National de Référence Mycoses et Antifongiques-Laboratoire Associé Asp-C, European Excellence Center for Medical Mycology (ECMM), Rennes, France; Universidade do Minho, Braga, Portugal

**Keywords:** COVID-19, CAPA, mycobiome, *Aspergillus*

## Abstract

Coronavirus disease 2019 (COVID-19)-associated pulmonary aspergillosis (CAPA) is a life-threatening complication in patients with severe COVID-19. Previously, acute respiratory distress syndrome in patients with COVID-19 has been associated with lung fungal dysbiosis, evidenced by reduced microbial diversity and *Candida* colonization. Increased fungal burden in the lungs of critically ill COVID-19 patients is linked to prolonged mechanical ventilation and increased mortality. However, specific mycobiome signatures associated with severe COVID-19 in the context of survival and antifungal drug prophylaxis have not yet been determined, and such knowledge could have an important impact on treatment. To understand the composition of the respiratory mycobiome in critically ill COVID-19 patients with and without CAPA and the impact of antifungal use in patient outcome, we performed a multinational study of 39 COVID-19 patients in intensive care units (ICUs). Respiratory mycobiome was profiled using internal transcribed spacer 1 sequencing, and *Aspergillus fumigatus* burden was further validated using quantitative PCR. Fungal communities were investigated using alpha diversity, beta diversity, taxa predominance, and taxa abundances. Respiratory mycobiomes of COVID-19 patients were dominated by *Candida* and *Aspergillus*. There was no significant association with corticosteroid use or CAPA diagnosis and respiratory fungal communities. Increased *A. fumigatus* burden was associated with mortality and, the use of azoles at ICU admission was linked with an absence of *A. fumigatus*. Our findings suggest that mold-active antifungal treatment at ICU admission may be linked with reduced *A. fumigatus-*associated mortality in severe COVID-19. However, further studies are warranted on this topic.

**IMPORTANCE:**

Invasive fungal infections are a serious complication affecting up to a third of patients with severe COVID-19. Nevertheless, our understanding of the fungal communities in the lungs during critically ill COVID-19 remains limited. Evidence suggests a higher fungal burden is associated with prolonged ventilation and higher mortality, although the particular organisms responsible for this link are unclear. Antifungal prophylaxis may be beneficial for reducing the burden of fungal co-infections in COVID-19 intensive care. However, the composition of the fungal microbiome in severe COVID-19 in relation to prophylactic antifungals, as well as how this is associated with survival outcomes, is yet to be studied. Our study provides insights into the lung fungal microbiome in severe COVID-19 and has found antifungal treatment to be associated with lower *Aspergillus fumigatus* burden and that higher levels of this pathogen are associated with mortality. Therefore, our study suggests mold-active antifungal prophylaxis may be beneficial in severe COVID-19.

## INTRODUCTION

Coronavirus disease 2019 (COVID-19) is a pulmonary disease caused by severe acute respiratory syndrome coronavirus 2. There have been over 700 million confirmed cases of COVID-19 since December 2019 with mortality of ~7 million (1). Around 5% of patients with COVID-19 require admission into the intensive care unit (ICU) (2, 3). Moreover, 50% of those patients need mechanical ventilation (4), thus increasing the risk of hospital-acquired pneumonia (5). The pulmonary microbiome and its associations with disease outcomes in COVID-19 patients have been explored since the beginning of the pandemic (6–8). However, our knowledge on the role of fungi in the pathophysiology of COVID-19 is limited. Specifically, the association between respiratory mycobiome composition and patient outcome and the interplay of antifungal use are yet to be investigated.

Mycobiome sequencing of the upper respiratory tract (nasopharyngeal swabs) suggests COVID-19 infection significantly reduces fungal diversity, with a higher abundance of *Alternaria* and *Cladosporium* spp. and a lower abundance of other taxa including *Candida* and *Aspergillus* (9). In the lower respiratory tract (tracheal aspirates), bacterial and fungal microbiome analyses of patients with severe COVID-19 have shown changes over time that might be linked to antimicrobial pressure (10). A variety of respiratory mycobiome clusters were identified, including those dominated by *Candida* and *Cladosporium*. Using 18S quantitative PCR (qPCR) in bronchoalveolar lavage (BAL) samples, studies have reported that critically ill COVID-19 patients with high fungal burdens are less likely to be liberated from mechanical ventilation (11). However, the taxa responsible for this outcome remain unclear. Lastly, mycobiome sequencing of BAL found COVID-19 patients with acute respiratory distress syndrome (ARDS) to be associated with reduced fungal diversity and an increase in *Candida* colonization (12). In patients without *Candida* colonization*,* an increased abundance of an unclassified Ascomycota species was identified.

COVID-19-associated pulmonary aspergillosis (CAPA) is an important complication of COVID-19, mainly described in critically ill patients. Multicenter cohort studies of CAPA conducted in the ICU setting report incidence rates varying between 10% and 15% (13–15). Nevertheless, mortality rates in patients with CAPA were double that observed in critically ill COVID-19 patients without CAPA (16, 17). Airway epithelial cell damage due to viral replication and COVID-19-associated downregulation of the interferon γ signaling pathway, aberrant immune responses due to ARDS, corticosteroids, azithromycin, or the use of immunomodulators have been linked with susceptibility to CAPA (18–21). With a view to investigating the impact of the respiratory mycobiome in the outcome of COVID-19, we performed a multinational mycobiome analysis of 39 respiratory samples from critically ill COVID-19 patients with and without CAPA.

## MATERIALS AND METHODS

### Study design, participants, and sample collection

This study was based on a multinational retrospective study on the prevalence of invasive pulmonary aspergillosis in critically ill COVID-19 patients in ICUs during 2020 (14). Inclusion criteria consisted of PCR-confirmed COVID-19 infection, bronchoscopy or tracheal aspiration performed during routine clinical investigations, and chest imaging available 7 days before or after respiratory samples were collected. Patients less than 18 years of age were excluded. Respiratory samples not passing quality control (described in the supplemental methods) were also excluded. Respiratory specimens obtained at ICU admission or during ICU stay were collected, aliquoted (at least 1 mL), and stored at −80°C. Criteria for defining aspergillosis were according to previous guidelines (22) with the following modifications: COVID-19 requiring ICU admission was included as an additional host factor; tracheal aspirates were equated to BAL fluid for microbiological tests; and serum and BAL galactomannan (GM) was added as entry criterion. For a summary of full patient demographics, see Table S1.

### Sample processing

BAL DNA was extracted using the cetyltrimethylammonium bromide method (23). Full details on sample processing are provided in the online supplement. Briefly, for mycobiome analysis, the internal transcribed spacer 1 (ITS1) region was amplified using Nextera XT compatible versions of ITS1 (24) and ITS2degen (a degenerate version of ITS2 primer) (see Table S1). The presence of *A. fumigatus* in respiratory samples was validated using a TaqMan probe assay targeting the ITS1 region (25). Full details are described in the supplemental methods.

### Data analysis

Raw sequence data have been deposited at the National Center for Biotechnology Information Sequence Read Archive under accession number PRJNA905224. The code used for analysis is available at https://github.com/Danweaver1/COVID_respiratory_mycobiome. Paired-end reads were subject to quality trimming at Q30 and a minimum length filter of 75 nucleotides using bbduk (26) (BBMap v.38.22). Primer sequences were removed using Cutadapt (27) (v.1.18). Reads were mapped to UNITE database (v.8.3) using bowtie2 (28) (v.2.3.5.1). Count data were further processed in R (v.4.1.3) using the following packages: phyloseq (29) (v.1.38.0), vegan (30) (v.2.5–7), DESeq2 (31) (v.1.34.0, stringr (32) (v.1.4.0), ggplot2 (33) (v.3.3.5), and tidyr (34) (v.1.2.0). Abundances were standardized to the median sequencing depth. Extremely low abundance taxa were removed by retaining only those occurring at >0.2% in any sample. DESeq2 was used to identify significantly differentially abundant taxa (*padj* <0.05 and base mean >500) (full results shown in Table S3). Differences in diversity [Shannon diversity, Chao1, and observed operational taxonomic units (OTUs)] were assessed using pairwise Wilcoxon rank-sum tests. Permutational multivariate analysis of variance test was used to assess differences in Bray-Curtis ordination.

## RESULTS

### Patient cohort

The respiratory mycobiome of 91 critically ill COVID-19 patients in the ICU was analyzed using ITS1 amplicon sequencing of BAL. Table S2 describes demographic and clinical characteristics of all patients. Samples from 39 patients harbored significant fungal communities which passed quality control (see Data Analysis section in the supplemental methods). Table 1 describes demographic and clinical characteristics of the 39 patients maintained in the mycobiome analysis, stratified by CAPA diagnosis. Of the 39 patients, 6 patients had a probable CAPA diagnosis (15%). Patients were from Genoa, Graz, and Rennes (64%, 26% and 10%, respectively). The mean ages of those with and without CAPA were 61 and 64, respectively. Most patients received systemic corticosteroids (83% of those with CAPA and 73% of those without CAPA). No patients diagnosed with CAPA received azole treatment at the time of ICU admission. Mortality at the end of follow-up in patients clinically diagnosed with CAPA was 33% (2/6), while mortality in patients without a CAPA diagnosis was also 33% (11/33).

**TABLE 1:** Clinical and demographic characteristics of patients with and without probable CAPA diagnosis^*a*^

Variable	Patients with CAPA (*N* = 6)	Patients without CAPA (*N* = 33)
Age		
Mean (SD)	61 (3.4)	64 (7.9)
Valid (missing)	6 (0)	32 (1)
Sex [% (*n*)]		
Male	100 (6)	79 (26)
Female	0 (0)	18 (6)
Missing	0 (0)	3 (1)
Ethnicity [% (*n*)]		
Caucasian	100 (6)	91.0 (30)
Other		6.1 (2)
Missing	0 (0)	3.0 (1)
BMI >30 [% (*n*)]		
Yes	50 (3)	18 (6)
No	50 (3)	79 (26)
Missing	0 (0)	3 (1)
Smoking [% (*n*)]		
Yes	33 (2)	12 (4)
No	67 (4)	85 (28)
Missing	0 (0)	3 (1)
Institution [% (*n*)]		
Graz	33 (2)	24 (8)
Genoa	17 (1)	73 (24)
Rennes	50 (3)	3 (1)
Hematology oncology [% (*n*)]		
Yes	17 (1)	9.1 (3)
No	83 (5)	88.0 (29)
Missing	0 (0)	3.0 (1)
Solid organ transplant [% (*n*)]		
Yes	17 (1)	3 (1)
No	83 (5)	94 (31)
Missing	0 (0)	3 (1)
Cardiovascular disease [% (*n*)]		
Yes	67 (4)	45 (15)
No	33 (2)	52 (17)
Missing	0 (0)	3 (1)
Pulmonary disease [% (*n*)]		
Yes	33 (2)	24 (8)
No	67 (4)	73 (24)
Missing	0 (0)	3 (1)
Diabetes mellitus [% (*n*)]		
Yes	17 (1)	9.1 (3)
No	83 (5)	88.0 (29)
Missing	0 (0)	3.0 (1)
Corticosteroids [% (*n*)]		
Yes	83 (5)	73 (24)
No	17 (1)	24 (8)
Missing	0 (0)	3 (1)
Tocilizumab [% (*n*)]		
Yes	17 (1)	6.1 (2)
No	83 (5)	91.0 (30)
Missing	0 (0)	3.0 (1)
Azithromycin [% (*n*)]		
Yes	33 (2)	33.0 (11)
No	67 (4)	61.0 (20)
Missing	0 (0)	6.1 (2)
Azole treatment at ICU admission [% (*n*)]		
Yes	0 (0)	15.0 (5)
No	100 (6)	6.1 (2)
Missing	0 (0)	79.0 (26)
Life support [% (*n*)]		
Mechanical	100 (6)	70.0 (23)
ECMO		3.0 (1)
Non-invasive		6.1 (2)
Mechanical and non-invasive		12.0 (4)
None		6.1 (2)
Missing	0 (0)	3.0 (1)
Duration ICU (days)		
Mean (SD)	27 (11)	32 (28)
Valid (missing)	6 (0)	32 (1)
Palliative (Day 28 or 32) [% (*n*)]		
Yes	67 (4)	48 (16)
No	33 (2)	48 (16)
Missing	0 (0)	3 (1)
Survival (at the end of follow-up) [% (*n*)]		
Yes	33 (2)	33 (11)
No	67 (4)	64 (21)
Missing	0 (0)	3 (1)
Days from ICU admission to CAPA		
Mean (SD)	6.2 (3.5)	–
BAL GM (ODI >1) [% (*n*)]		
Positive	83 (5)	9.1 (3)
Negative	17 (1)	82.0 (27)
Missing	0 (0)	9.1 (3)
BAL PCR [% (*n*)]		
Positive	50 (3)	0 (0)
Negative	0 (0)	58 (19)
Missing	50 (3)	42 (14)
BAL culture [% (*n*)]		
Positive	50 (3)	0 (0)
Negative	50 (3)	97 (32)
Missing	0 (0)	3 (1)
BAL LFD [% (*n*)]		
Positive	17 (1)	6.1 (2)
Negative	0 (0)	9.1 (3)
Missing	83 (5)	85.0 (28)
Tracheal aspirate GM [% (*n*)]		
Positive	0 (0)	3 (1)
Negative	0 (0)	3% (1)
Missing	100 (6)	94 (31)
Tracheal aspirate PCR [% (*n*)]		
Positive	33 (2)	0 (0)
Negative	17 (1)	21 (7)
Missing	50 (3)	79 (26)
Tracheal aspirate culture [% (*n*)]		
Positive	17 (1)	0 (0)
Negative	33 (2)	3 (1)
Missing	50 (3)	97 (32)
Serum GM (>0. 5) [% (*n*)]		
Positive	33 (2)	0 (0)
Negative	50 (3)	42 (14)
Missing	17 (1)	58 (19)
Bronchial aspirate culture [% (*n*)]		
Positive	17 (1)	0 (0)
Negative	33% (2)	21 (7)
Missing	50 (3)	79 (26)

^*a*^ BAL, bronchoalveolar lavage; BMI, body mass index; CAPA, COVID-19-associated pulmonary aspergillosis; ECMO, extracorporeal membrane oxygenation; GM, galactomannan; ICU, intensive care unit; LFD, lateral flow device.

### *Candida* and *Aspergillus* spp. dominate respiratory mycobiomes in critically ill COVID-19 patients

**Fig 1:**
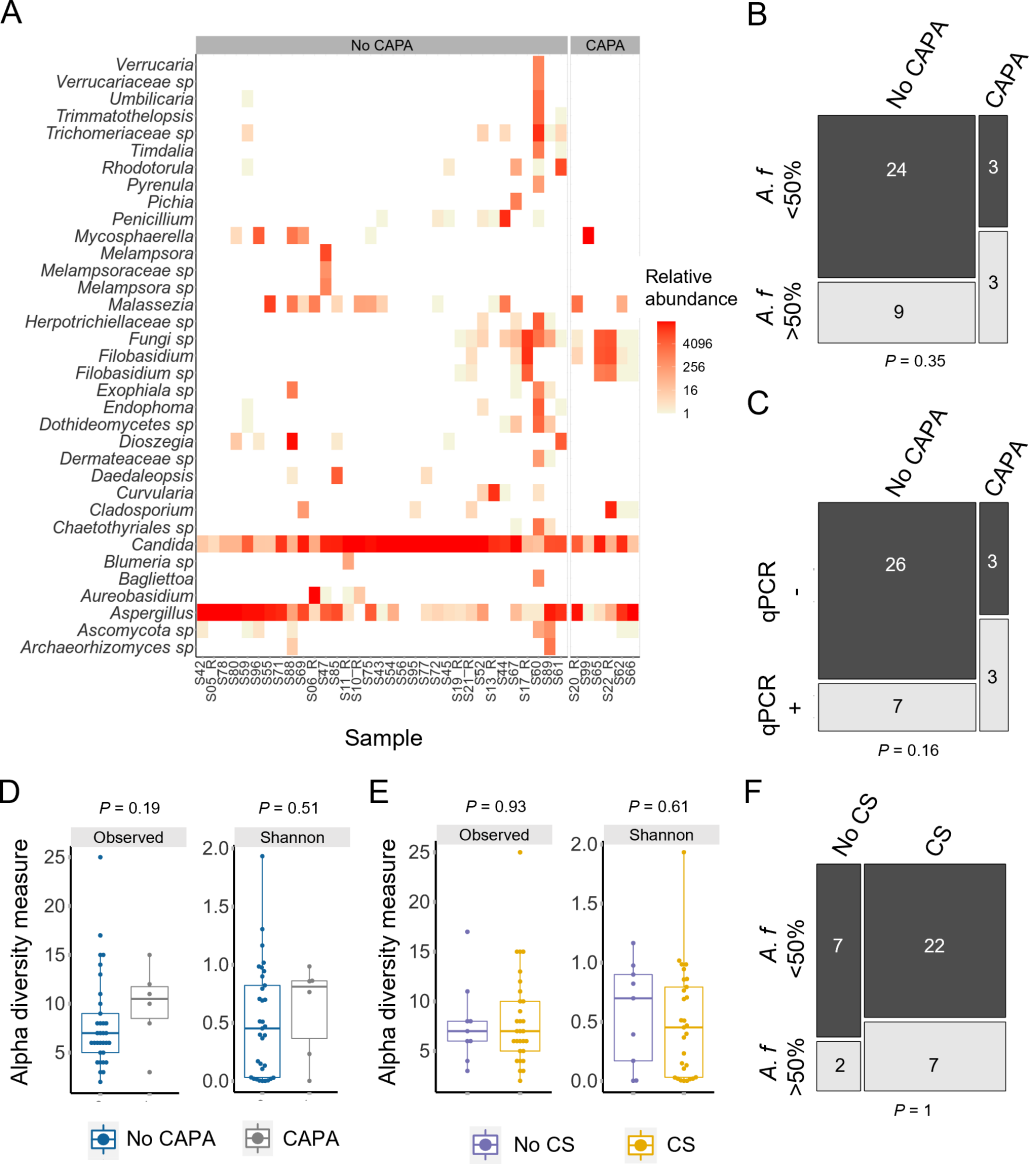
*Aspergillus* and *Candida* spp. dominate the respiratory mycobiome in critically ill COVID-19 patients. (**A**) *Aspergillus* and *Candida* were the main genera observed in the lungs from COVID-19 patients included in the study (*n* = 39). Samples are grouped based on CAPA status. (**B**) *A. fumigatus w*as the predominant species in the mycobiomes of 50% of patients with CAPA, compared to 27% of those without CAPA. (**C**) Fifty percent of CAPA patients were *A. fumigatus* positive by species-specific qPCR, compared to 21% of those without CAPA. (**D**) Alpha diversity measures (observed OTUs and Shannon diversity) trended toward higher diversity in CAPA patients (**E**) Corticosteroid treatment caused no apparent effect on alpha diversity as measured by observed OTUs or Shannon diversity. (**F**) *A. fumigatus w*as the predominant species in the mycobiomes of 24% of patients receiving corticosteroids, compared to 22% of those without corticosteroids. Hypothesis testing was applied using Wilcoxon’s rank-sum tests (**D,E**) or Fisher’s exact tests (**B,C,F**). Boxplot data represent median and interquartile range. CAPA, COVID-19-associated pulmonary aspergillosis; CS, corticosteroid.

The median read count per sample for the 39 samples that passed quality control was 57,913 (range 6,970–342,640). There were 36 genera in total and a median of 5 genera per sample (range 1–22). There was no significant clustering between sample batches (Fig. S1), suggesting that sample processing had no impact on mycobiome communities. Mycobiomes predominantly consisted of *Candida* and *Aspergillus* (Fig. 1A). *Candida albicans*, *Aspergillus fumigatus*, and *Candida parapsilosis* were the most abundant species (Fig. S2A and B). Notably, *Candida auris* was identified in two patients (Fig. S2A).

### No significant correlation is found between mycobiome communities and CAPA status or corticosteroid use

In our study, higher median *A. fumigatus* levels were observed in CAPA when assessed by read count (~16,700 vs 35) and *A. fumigatus*-specific qPCR (0.3 vs 0 genome equivalents) (Fig. S3A and B), but there was an overlap between the patient groups and statistical significance was not reached. *A. fumigatus* burden in patients without a CAPA diagnosis was varied. Some patients had little to no *A. fumigatus*, and others contained a particularly high burden (Fig. 1A; Fig. S3A and B). As *A. fumigatus* levels appeared to be bimodal, we dichotomized these data into two groups to assess either the predominance of *A. fumigatus* (present at over 50% of a mycobiome sample) or high *A. fumigatus* burden (qPCR positive at over 0.1 haploid genome equivalents). Analyzing the data in this manner also found no significant difference between *A. fumigatus* predominance (Fig. 1B) or high burden (Fig. 1C) in patients with and without a CAPA diagnosis. Furthermore, species differential abundance analysis did not find significant differences between *A. fumigatus* levels based on CAPA status. Instead, *Cladosporium delicatulum*, *Mycosphaerella tassiana*, and *Filobasidium magnum* were found to be at higher abundance in CAPA patients (Fig. S6). In addition, probable CAPA patients had higher median alpha diversity; however, this difference was not significant, and no significant effect on beta diversity was observed (Fig. 1D; Fig. S3C).

Corticosteroid use resulted in lower median alpha diversity (Shannon diversity only); however, this difference was not significant (Fig. 1E). Median levels of *A. fumigatus* were lower in the corticosteroid treated group as assessed by sequencing (8 vs ~5,800 reads) and qPCR (0 vs 0.1 genome equivalents) (Fig.S3D and E). However, patients on corticosteroids were highly heterogenous in terms of *A. fumigatus* abundance. There was no significant difference between *A. fumigatus* predominance (Fig. 1F) or high burden (Fig. S3F) in patients with and without corticosteroid treatment. Species differential abundance analysis found no significant differences between corticosteroid usage. In addition, corticosteroid use had no significant impact on beta diversity (Fig. S3G).

### Increased *A. fumigatus* burden is associated with mortality

**Fig 2:**
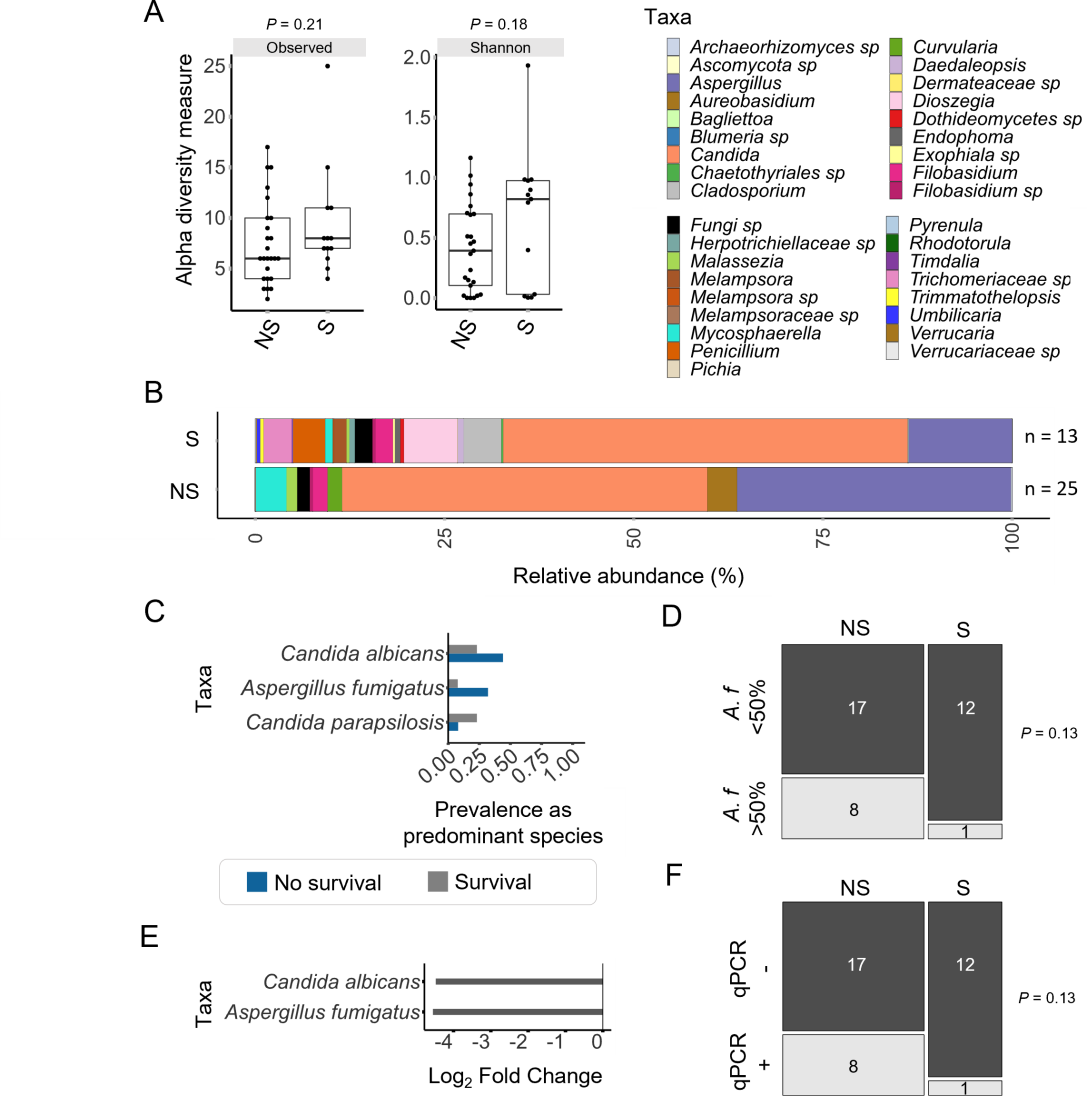
A higher *A. fumigatus* burden is associated with mortality in critically ill COVID-19 patients. (**A**) Alpha diversity measures (observed OTUs and Shannon diversity) trended toward higher diversity in critically ill COVID-19 patients who survived. Data represent median and interquartile range. (**B**) At the genus level, pooled relative abundance mycobiome data from patients who survived (*n* = 13) indicated a lower proportion of *Aspergillus* and an apparent increase in the number of observed taxa overall. (**C**) *C. albicans* and *A. fumigatus* were prevalent as the predominant species in a mycobiome in more patients who did not survive (blue, prevalence of 0.44 and 0.32, respectively) than those who did survive (gray, prevalence of 0.32 and 0.08, respectively). Taxa were counted if present at over 50% of the total counts, and only taxa found in at least 10% of the samples of either group are shown. (**D**) *A. fumigatus* was the predominant species in 8 of 25 (32%) patients who did not survive, compared to 1 of 13 (8%) of patients who did survive. (**E**) Analysis using DESeq2 identified *A. fumigatus* and *C. albicans* to be at significantly lower abundance in patients who survived. (**F**) Thirty-two percent of patients who did not survive were *A. fumigatus* positive by species-specific qPCR, compared to 7% of those which survived. Hypothesis testing was applied using Wilcoxon rank-sum tests (**A**), Fisher’s exact tests (**D and F**), or DESeq2 (**E**). NS, no survival; S, survival.

The mycobiome of surviving individuals showed a trend toward higher median alpha diversity (Fig. 2A). Grouped mean abundances indicated a lower level of *Aspergillus* was present upon survival (Fig. 2B). At the individual sample level, many mycobiomes of non-surviving patients were predominated by *Aspergillus* (Fig. S4). Species predominance analysis suggested that this difference was due to *A. fumigatus*, with 32% (8 of 25) of non-surviving patients’ mycobiomes being dominated by this species compared to only 8% (1 of 13) of patients who survived (Fig. 2C and D). Furthermore, mycobiome differential abundance analysis found *A. fumigatus* and *C. albicans* to be significantly less abundant upon survival, with log fold change values of −4.3 and −4.6, respectively (*padj* <0.05) (Fig. 2E). Quantitative PCR data also showed 32% of patients who did not survive displayed a high burden of *A. fumigatus* compared to only 8% of surviving patients (Fig. 2F). All datapoints for *A. fumigatus* relative abundance and qPCR burden are shown in Fig. S5A and B. BAL GM index values were not significantly different between the patient groups, with an outlier in the survival group having a very high index (Fig.S5C).

### Azole treatment at intensive care unit admission is associated with reduced *A. fumigatus* burden in critically ill COVID-19 patients and COVID-19 survival

**Fig 3:**
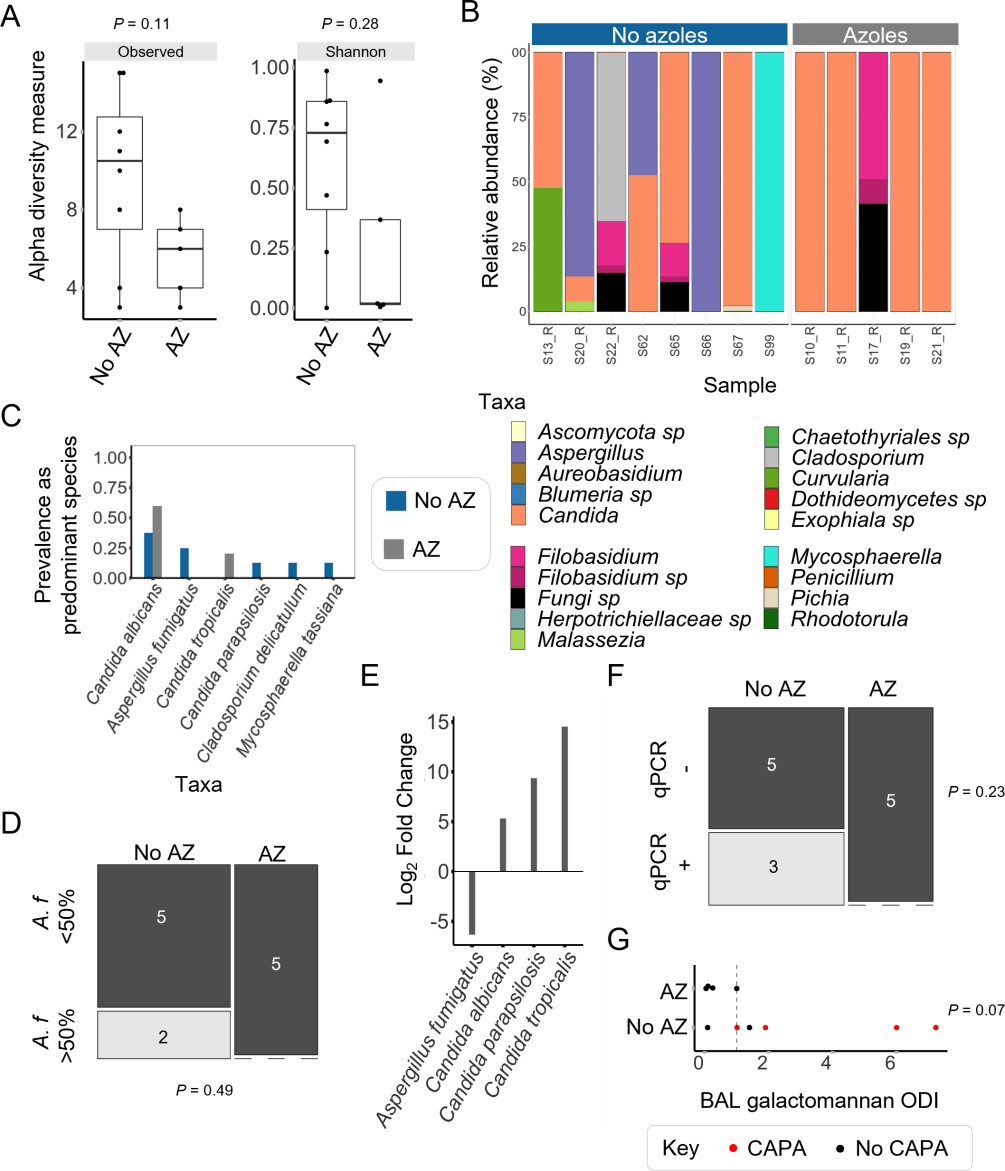
*A. fumigatus* is associated with the absence of azole treatment at intensive care unit admission in critically ill COVID-19 patients. Data shown here are for a subset of patients (*n* = 13) for which azole treatment information was available. (**A**) Alpha diversity measures (observed OTUs and Shannon diversity) trended higher diversity in COVID-19 patients without azoles at ICU admission. (**B**) At the genus level, BAL ITS1 mycobiomes from COVID-19 patients who received azoles (*n* = 5) displayed an absence of *Aspergillus*, whereas three of eight patients not receiving azoles harbored *Aspergillus*. For simplicity, genus-level data are shown here. The corresponding species data are shown in Fig. S7A. (**C**) *C. albicans* was prevalent as the predominant species of a mycobiome in more patients who received azoles (gray, prevalence of 0.6) than those who did not receive azoles (blue, prevalence of 0.38). *A. fumigatus* was prevalent as the predominant species in 25% of patients receiving azoles. *A. fumigatus* was not prevalent in any patients who received azoles. Taxa were counted if present at over 50% of total counts, and only taxa found in at least 10% of samples of either group are shown. (**D**) *A. fumigatus* was the predominant species in two of eight (25%) patients who did not survive, compared to none of the patients which did survive. (**E**) Analysis using DESeq2 identified *A. fumigatus* to be at significantly lower abundance in patients who received azoles. *C. albicans*, *C. parapsilosis*, and *C. tropicalis* were all at significantly higher abundance in patients receiving azoles. (**F**) Thirty eight percent (three of eight) of patients who did not receive azoles were *A. fumigatus* positive by species-specific qPCR, compared to no patients who did receive azoles. (**G**) All patients receiving azoles were BAL galactomannan negative (ODI of 1 or lower). Two-thirds (four of six) of patients not receiving antifungals were galactomannan positive. Hypothesis testing was applied using Wilcoxon rank-sum tests (**A and G**), Fisher’s exact tests (**D and F**), or DESeq2 (**E**). AZ, azole; ODI, optical density index.

For a subset of patients (13 of 39), data were available regarding the use of azole treatment, and subsequent analyses are on this group of patients only. Use of azole treatment at ICU admission in critically ill COVID-19 patients resulted in significantly reduced alpha diversity when analyzing raw mycobiome data. Upon removal of very rare taxa, there was a trend toward reduced median alpha diversity upon azole treatment (Fig. 3A). There was a lack of *Aspergillus* in the mycobiomes of patients with azole treatment, and *Aspergillus* was present at a considerable relative abundance in ~38% (three of eight) of patients receiving azoles (Fig. 3B). At the species level, *A. fumigatus* was the predominant species in 25% of patients without azole treatment, whereas this species was not detectable in patients receiving treatment (Fig. 3C and D). Furthermore, differential abundance analysis of mycobiome data found a significant reduction of *A. fumigatus* in patients who underwent azole treatment (log_2_ fold change (LFC) −6.3, *padj* 0.04) (Fig. 3E). This analysis also found *Candida albicans*, *Candida parapsilosis*, and *Candida tropicalis* had significantly higher abundance in COVID-19 patients receiving azole treatment (LFC 5.3, 9.4, and 14.5, respectively). qPCR data found 38% of patients which did not receive azoles displayed a high burden of *A. fumigatus* compared to no patients on azole treatment (Fig. 2F). Datapoints for *A. fumigatus* relative abundance and qPCR burden with and without azole treatment at ICU admission are shown in Fig. S5D and E. BAL galactomannan index was not statistically different between patients with or without azole treatment. However, all individuals receiving treatment were GM negative, whereas only half of the patients without treatment were GM negative (Fig. 3G).

**Fig 4:**
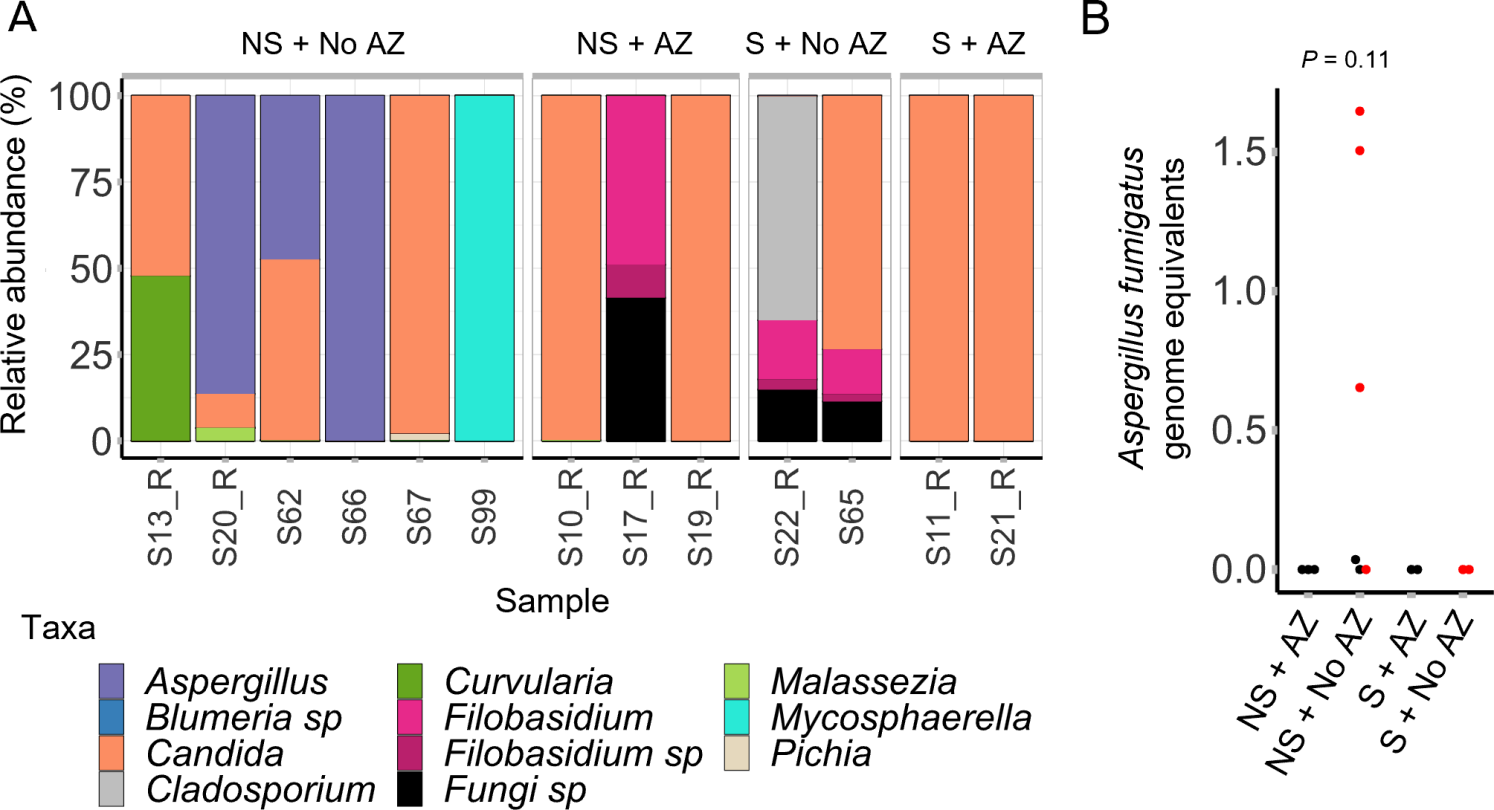
*A. fumigatus* is associated with mortality in patients with COVID-19 who have not received azole treatment at the intensive care unit admission. Data shown here are for a subset of patients (*n* = 13) for which azole treatment information was available. (**A**) When combining the use of azoles at ICU and survival outcomes, *Aspergillus* was found in the BAL ITS1 mycobiomes from 50% of patients who neither received azoles nor survived (three of six). No *Aspergillus* was present in samples from the remaining three patient groups. For simplicity, genus-level data are shown here. The corresponding species data are shown in Fig. S7B (**B**) *A. fumigatus* levels (measured by qPCR) did not differ significantly between groups (Wilcoxon rank-sum test). However, *A. fumigatus* burden was observed only in the patient group which neither survived nor received azole treatment at ICU admission. AZ, azole; NS, no survival; S, survival.

Our findings suggest an association between *A. fumigatus* abundance and mortality in critically ill COVID-19 patients and that azole treatment at ICU significantly reduces *A. fumigatus* levels. Therefore, we combined these factors to assess the association between *A. fumigatus* and survival outcomes, depending on the presence or absence of azole treatment. It was apparent that *Aspergillus* was associated with mortality only in COVID-19 patients who had not received azole treatment (Fig. 4A). Presence of *A. fumigatus* in only those patients who did not survive or receive azole treatment was confirmed by qPCR (Fig. 4B). Therefore, these findings suggest azole treatment at ICU admission may have been protective against *A. fumigatus-*associated mortality in this patient cohort with severe COVID-19.

## DISCUSSION

This multinational study found that higher *Aspergillus fumigatus* levels in critically ill COVID-19 patients were associated with increased mortality. In addition, the association of *A. fumigatus* with mortality was found only in patients who did not receive azole treatment at ICU admission, suggesting that the use of prophylactic mold-active antifungals in patients with severe COVID-19 is potentially valuable for the reduction of *A. fumigatus*-associated mortality in this cohort.

The respiratory mycobiomes of critically ill COVID-19 patients described here were dominated by *Candida* and *Aspergillus*. Previous studies using similar patient groups have also reported lung fungal communities to contain *Candida* and *Aspergillus*; however, the mycobiomes identified were largely dominated by *Candida* (10, 12). However, one study identified a significant increase in unidentified Ascomycota species in patients without *Candida* colonization (12). Due to the reported incidence of CAPA in severe COVID-19, the authors hypothesized that *Aspergillus* could be present in these patients and, although COVID mycobiome data were mostly negative for *Aspergillus* (excluding one patient), the presence of *Aspergillus* was confirmed by PCR in follow-up BAL samples in over 20% of patients. As noted by the authors, the lack of *Aspergillus* identification in the mycobiome analysis of this study could have been due to a variety of factors such as changes in *Aspergillus* load during the development of CAPA and the occurrence of *Candida* colonization obscuring the detection of *Aspergillus* by sequencing (but not targeted PCR). In addition, data analysis method may influence the taxa that are identified. In this study, we aligned sequencing reads directly to the UNITE database using a short read alignment tool. Unlike ASV/OTU clustering methods, this method does not require paired-end reads to overlap and be merged in the analysis. This was particularly important in this study as we employed the iSeq100, which produces relatively short reads, and certain fungal taxa have relatively large ITS1 amplicons. Our method may have a different bias compared to commonly used OTU/ASV clustering methods such as QIIME2 and DADA2. Direct alignment is more likely to accurately identify taxa to the species level; however, it may be less effective at identifying closely related taxa that are not present in the database. We performed Basic Local Alignment Search Tool (BLAST) searches to validate the identifications of clinically relevant taxa such as *A. fumigatus* and *C. albicans*. Therefore, although our method may be less suitable for exploratory community analyses, it is suitable for a more clinically focused analysis.

A recent report suggests higher fungal burden in the lung microbiota of patients with proven/probable CAPA (11). However, a considerable overlap in fungal burdens between those with and without CAPA was noted. Our study found CAPA patients had higher median levels of *A. fumigatus* and trended toward higher fungal diversity. However, these findings did not meet statistical significance, which may have been driven by the low sample size (*n* = 6 in the CAPA group). Some patients within the non-CAPA group harbored considerable levels of *A. fumigatus*. It is known that high *Aspergillus* burdens can be found in the lungs of healthy small mammals and humans (35, 36). These observations suggest that if a sufficient level of *Aspergillus* is present in the lung, other factors such as disease susceptibility or strain virulence in the context of CAPA may be more important than burden in the outcome of infection. This study was limited to one sample time point, and it would be interesting to assess how *Aspergillus* burden changes during CAPA or COVID-19 infection.

It has been suggested that corticosteroid treatment increases lung fungal burden (particularly *A. fumigatus*) (37) and lowers Shannon diversity (38) in asthma. In contrast, no significant differences were found between mycobiome diversity or taxa abundance in respiratory fungal communities of COPD patients with or without inhaled corticosteroid treatment (39). Another recent study found that alterations in the airway mycobiome in COPD were not significantly affected by corticosteroid use (40). Large cohort studies suggest systemic corticosteroids are a risk factor for CAPA (15, 18). However, there are no specific reports of the influence corticosteroid use has on lung fungal communities in COVID-19. In our study, patients receiving corticosteroids displayed lower median levels of *A. fumigatus* and lower fungal diversity compared to those not receiving corticosteroids. However, these differences did not meet statistical significance. As most (~74%) patients received corticosteroids in this cohort, this may have contributed to low power in these statistical comparisons, warranting further data on this topic.

A high fungal burden has previously been associated with a lower likelihood of release from mechanical ventilation and increased mortality risk in patients with severe COVID-19 (11). However, as this study utilized pan-fungal qPCR to identify burden, there was no indication of the specific fungal taxa responsible for this association. Our findings suggest that higher levels of *A. fumigatus* are associated with increased mortality in severe COVID-19. There are no previous reports on the impact of antifungal use on the respiratory mycobiome in COVID-19 patients. In this study, the use of azoles at ICU admission was associated with an absence of *A. fumigatus* and appeared protective against *A. fumigatus-*associated mortality.

Our study investigated the composition of respiratory fungal communities in critically ill COVID-19 patients with and without CAPA. *Candida* and *Aspergillus* were predominant in the respiratory communities. CAPA diagnosis was associated with higher median *A. fumigatus* level and fungal diversity, and a higher prevalence of *A. fumigatus* was associated with mortality and a lack of azole treatment at ICU admission. Our data suggest that the potential use of prophylactic antifungals (with anti-*Aspergillus* activity) in seriously ill COVID-19 patients is worthy of further consideration for the possible prevention of *A. fumigatus*-associated mortality. However, a limitation of this study is the small number of patients included, particularly the low number of CAPA cases. In addition, incomplete clinical data with respect to azole use reduced the sample sizes for this comparison, which may have resulted in low power for these statistical tests. Therefore, a study of a larger cohort would be valuable to improve our understanding of the association between prophylactic azole use, the presence of *A. fumigatus* in the respiratory mycobiome, and patient outcome in COVID-19 critical care.

## AFTER EPUB

[This article was published on 19 December 2024 with missing funding information. The funding information was updated in the current version, posted on 24 January 2025.]

## SUPPLEMENTAL MATERIAL

Supplemental material spectrum.04110-23-s0001.docx Supplemental figures, tables, and methods.

ASM does not own the copyrights to Supplemental Material that may be linked to, or accessed through, an article. The authors have granted ASM a non-exclusive, world-wide license to publish the Supplemental Material files. Please contact the corresponding author directly for reuse.
