# Supplementary material for: Mycobiome analyses of critically ill COVID-19 patients
Source: Microbiol Spectr. 2024 Dec 19;13(2):e04110-23. doi: 10.1128/spectrum.04110-23 (PMC11792475; doi:10.1128/spectrum.04110-23)
Supplement: Supplemental material — Supplemental figures, tables, and methods. [file spectrum.04110-23-s0001.docx]

# Supplementary data


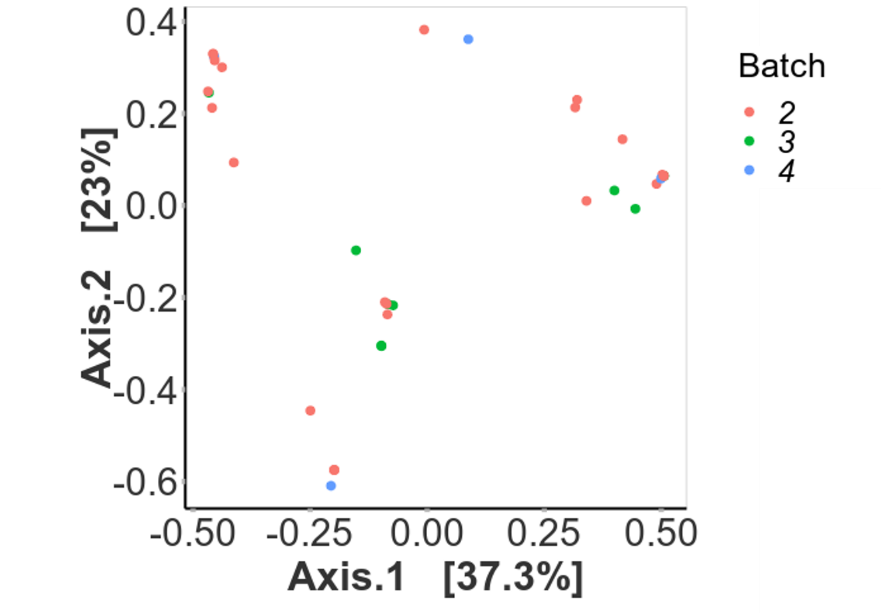


**Fig. E1. Mycobiome Bray-Curtis ordination by sample batch.**


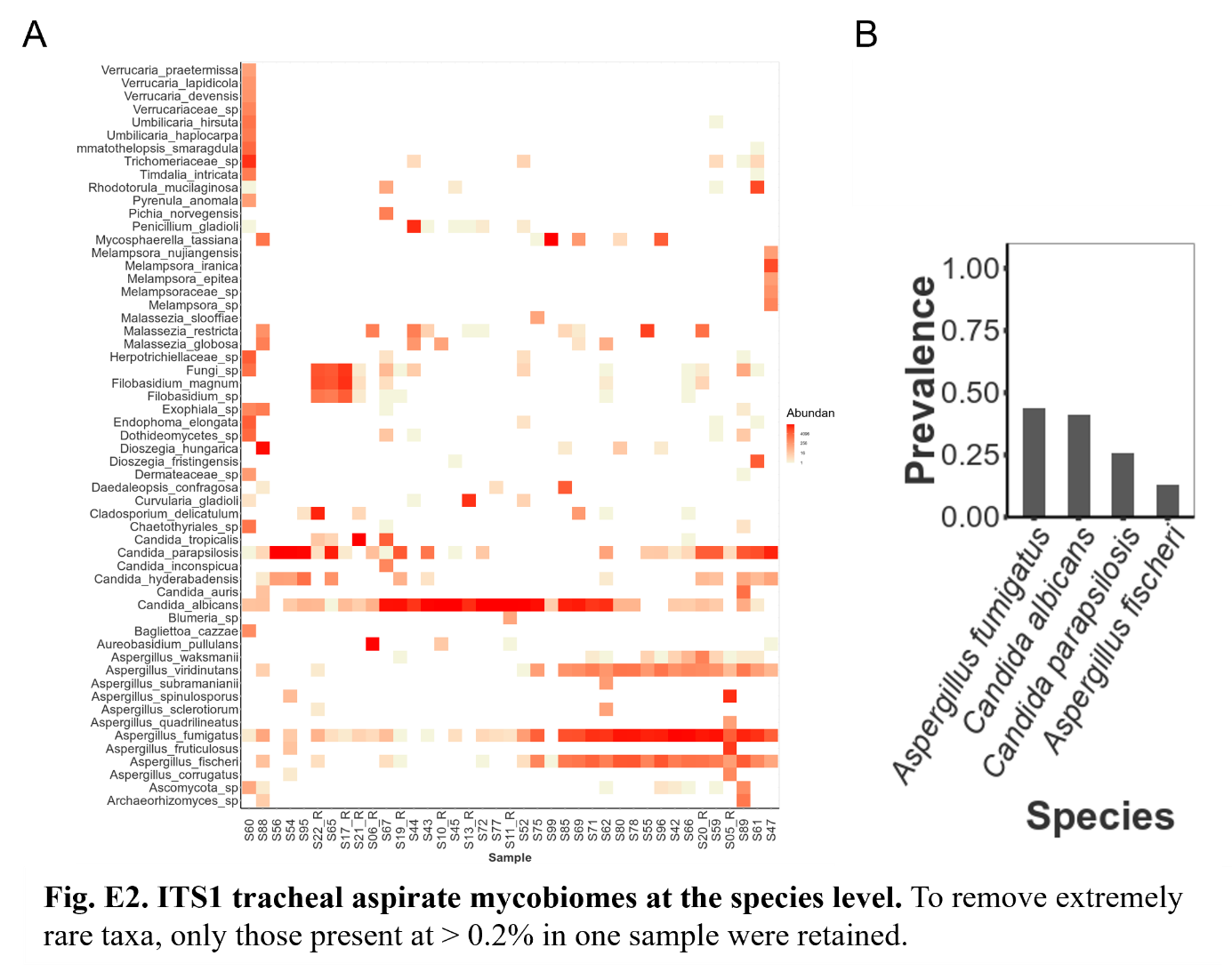


**Fig. E2. ITS1 respiratory mycobiomes at the species level.** (A) Heatmap of fungal species identified in all samples. To remove extremely rare taxa, only those present at > 0.2% in one sample were retained. (B) *A. fumigatus* and *C. albicans* are the most prevalent species. Taxa were counted if present at over 5% in a sample, and only taxa found in at least 10% of samples of either group are shown.


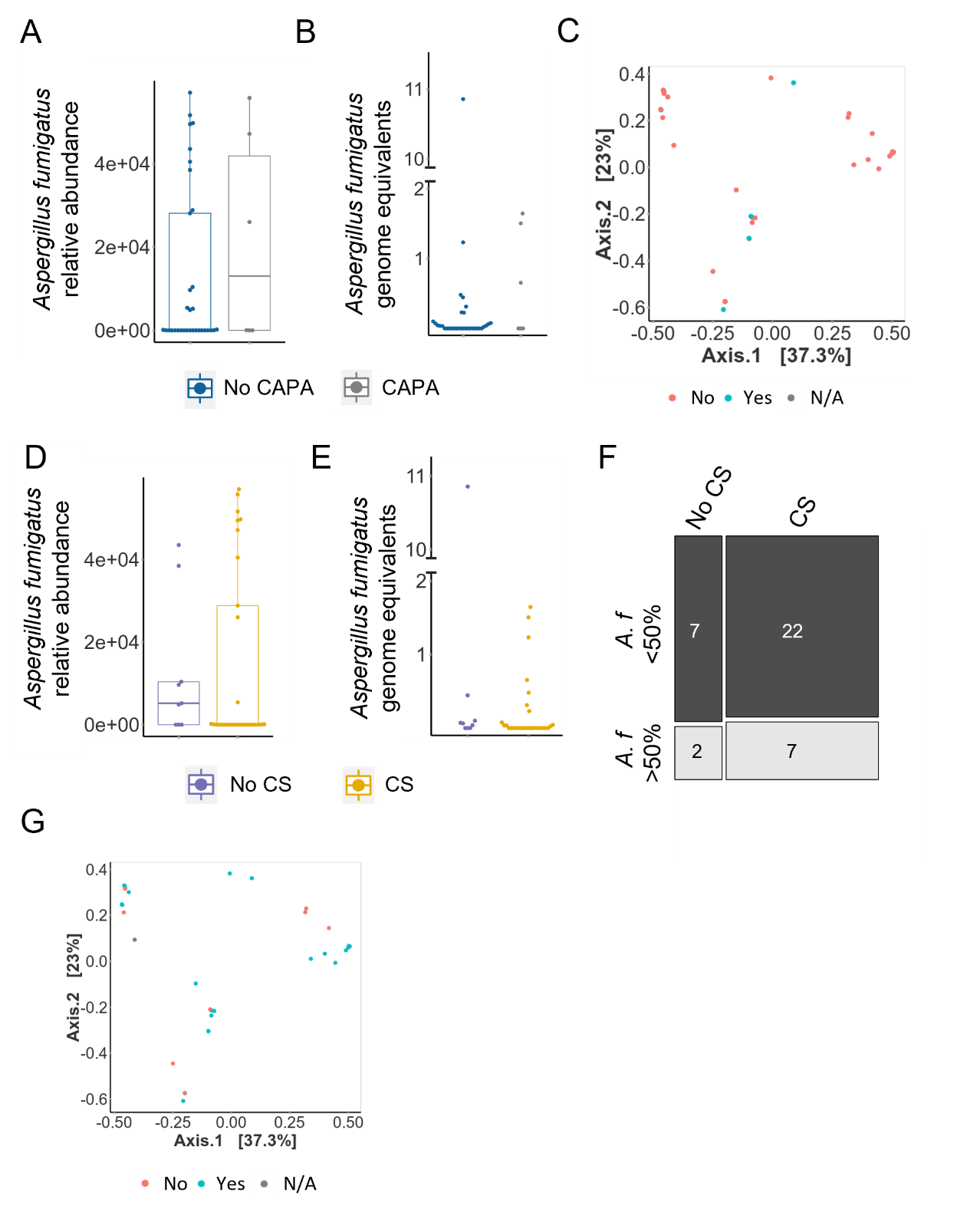


Fig. E3. CAPA diagnosis and corticosteroid use had no significant impact on fungal burden, *Aspergillus fumigatus* levels or beta diversity. (A) Relative abundance of *A. fumigatus* in mycobiomes of those with and without CAPA. (B) *A. fumigatus* levels in those with and without CAPA measured by qPCR. (C) Beta diversity and CAPA status. (D) Relative abundance of *A. fumigatus* in mycobiomes of those with and without corticosteroid use. (E) *A. fumigatus* levels in those with and without corticosteroid use as measured by qPCR. (F) Dichotomised data for samples positive/negative for high *A. fumigatus* burden by qPCR in patients with or without corticosteroids*.* (G) Beta diversity and corticosteroid use. Boxplot data represent median and interquartile range. CS; corticosteroid use. CAPA; COVID-associated pulmonary aspergillosis.


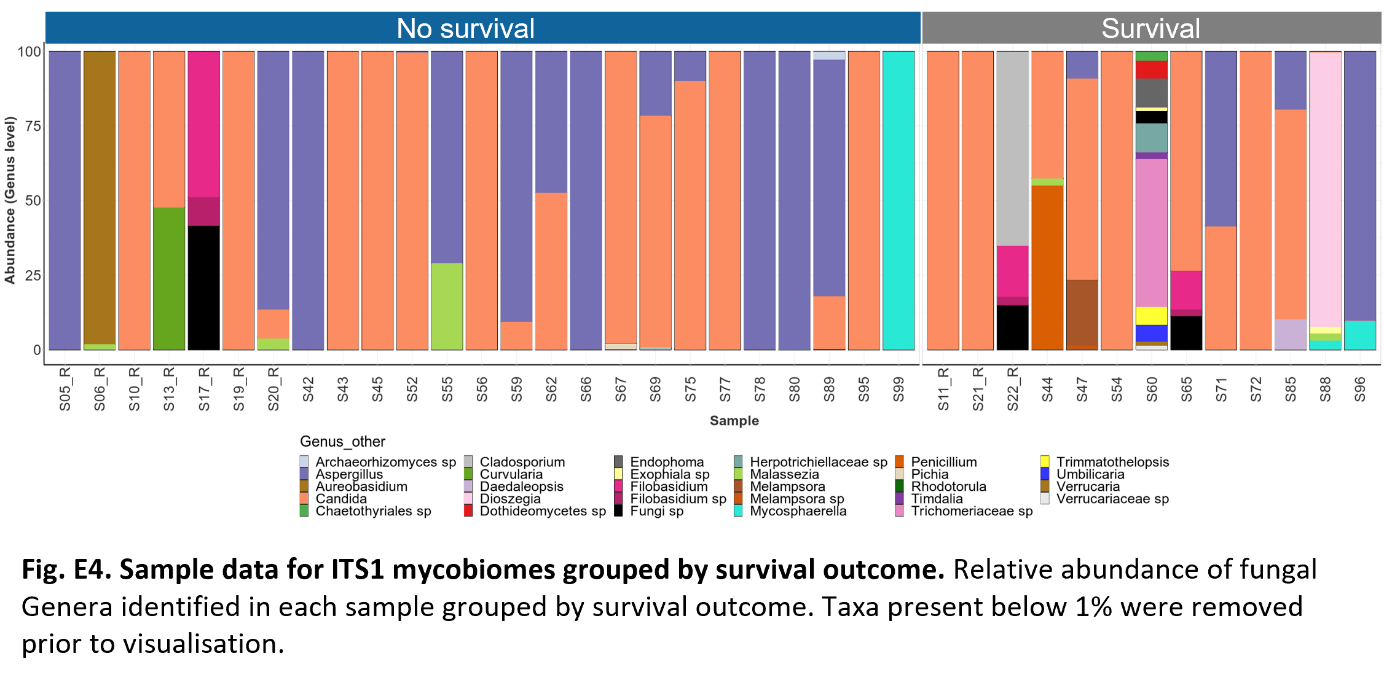


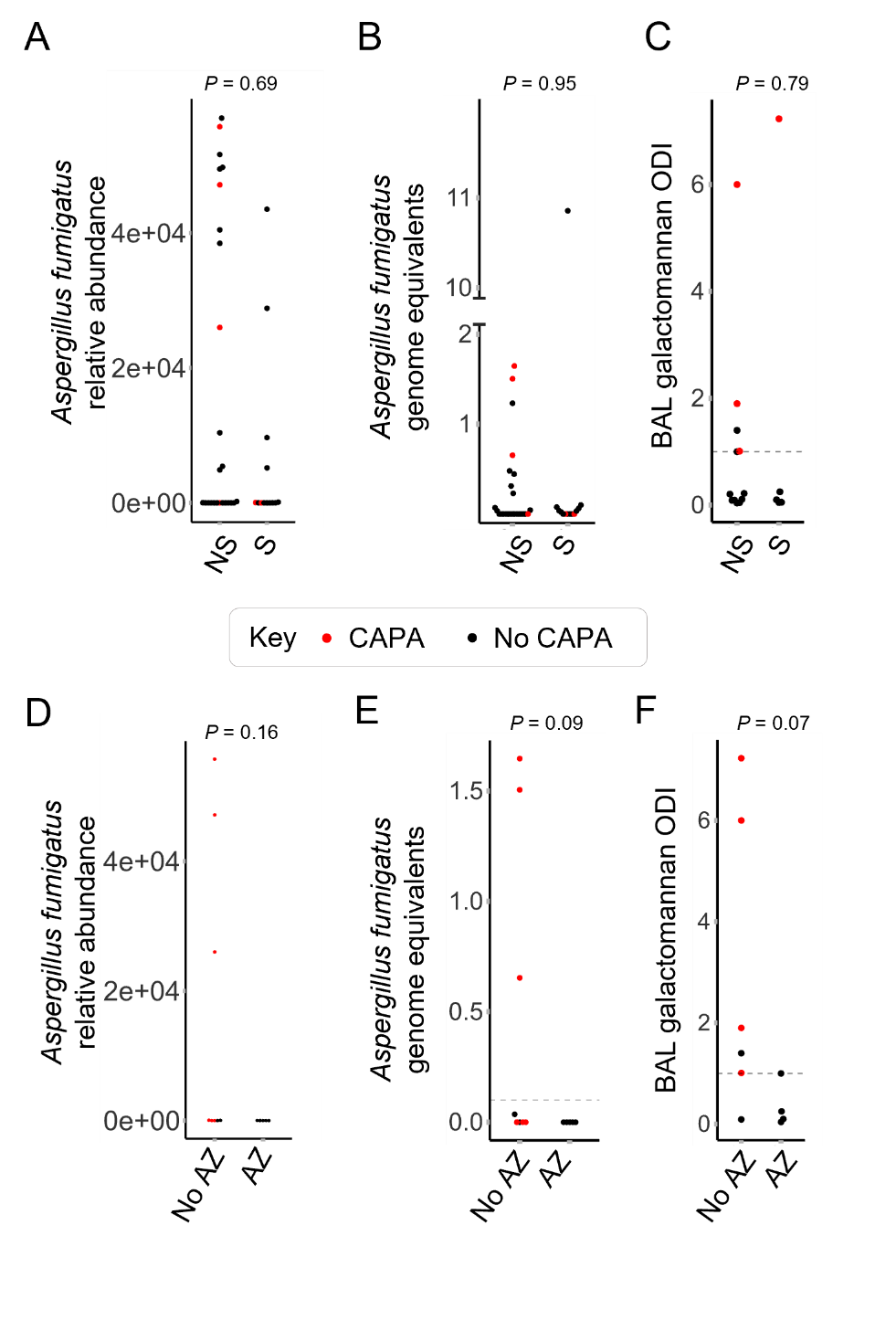
 **Fig. E5. Extended data for *Aspergillus fumigatus* levels in respiratory mycobiomes and BAL galactomannan.** (A) *Aspergillus fumigatus* relative abundance in patients grouped by survival outcome. (B) *A. fumigatus* burden measured by qPCR in patients which did not survive appeared biomodal, with some patients exhibiting a high burden (>0.1 genome equivalents). Excluding one outlier with an extremely high burden, all patients which survived displayed a low burden of 0.1 or below. (C) BAL galactomannan levels of patients grouped by survival outcome. (D) *Aspergillus fumigatus* relative abundance in patients grouped by azole treatment at ICU admission. (E) *A. fumigatus* burden measured by qPCR in patients which did or did not receive azole treatment at ICU admission. (F) All patients receiving azole treatment were BAL galactomannan negative (1 or lower). Half of patients not receiving azole treatment were galactomannan positive. CAPA: COVID-associated pulmonary aspergillosis. AZ: Azole treatment; No AZ: No azole treatment; S: Survival; NS: No survival.


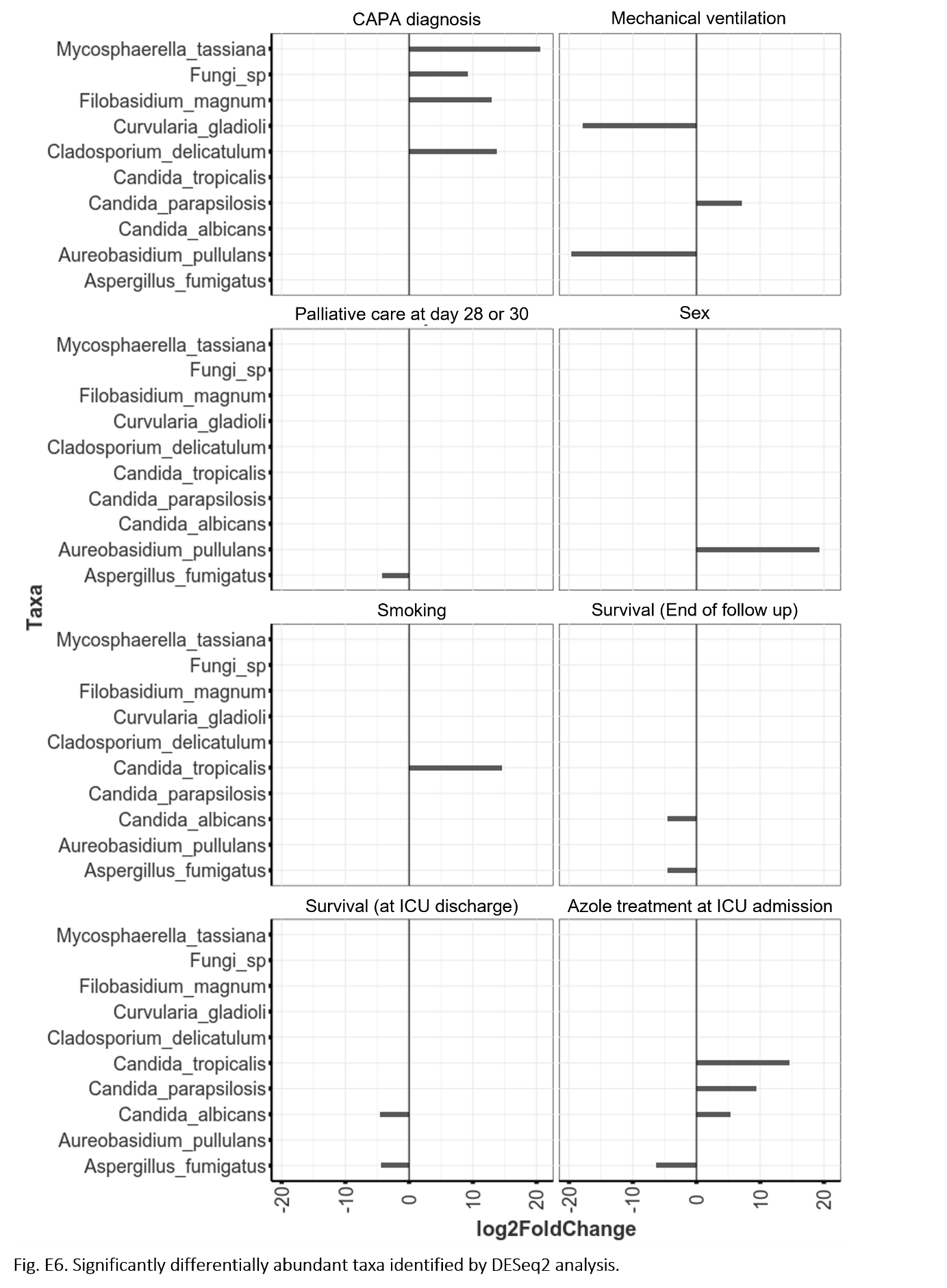


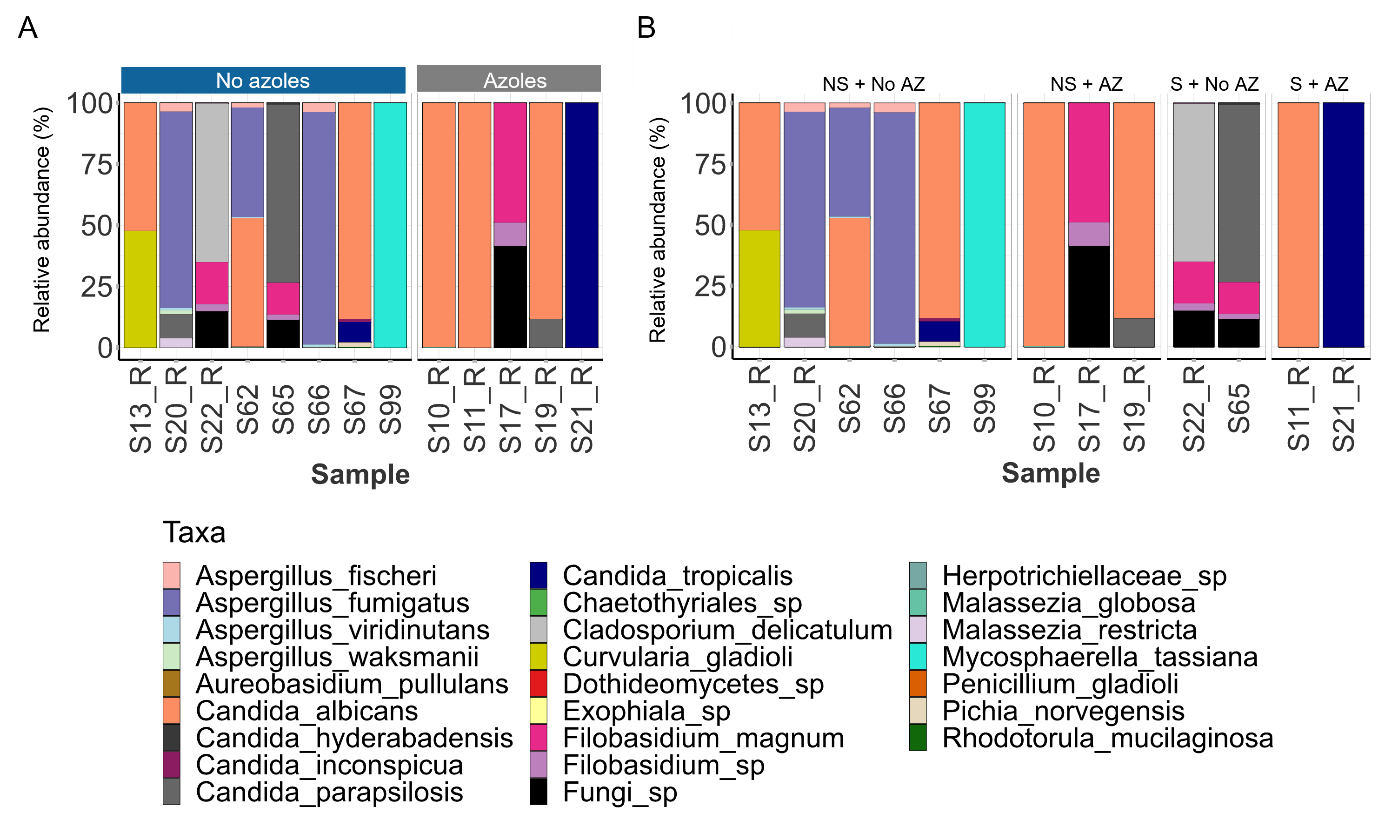


**Fig. E7. Species data for mycobiome associations with azole treatment and mortality** (A) At the species level, BAL ITS1 mycobiomes from COVID-19 patients which received azoles (n =5) display an absence of *A. fumigatus,* whereas 3/8 patients not receiving azoles harboured *A. fumigatus.* (B) When combining the use of azoles at ICU and survival outcomes, *A. fumigatus* was found in the BAL ITS1 mycobiomes from 50% of patients which did not receive azoles or survive (3/6). No *A. fumigatus* was present in samples from the remaining three patient groups.

**Table E1. Primer table.** Nextera XT adaptors are underlined. FunITS2-Ill is a Nextera XT-compatible, modified version of ITS2^25^ (modified bases are indicated in bold).

| **Primer name** | **Sequence (5’-3’)** | **Reference** |
| --- | --- | --- |
| FunITS1-Ill | TCGTCGGCAGCGTCAGATGTGTATAAGAGACAGTCCGTAGGTGAACCTGCGG | Modified from 25 |
| FunITS2-Ill | GTCTCGTGGGCTCGGAGATGTGTATAAGAGACAGGC **Y**RCGTTCTTCATCGA**Y**GC | Modified from 25 |

**Table E2. Full clinical patient demographics**

| Characteristics | N=39 |
| --- | --- |
| Age |  |
| Mean (SD) | 64 (7.4) |
| valid (missing) | 38 (1) |
| Sex |  |
| male | 82% (32) |
| female | 15% (6) |
| missing | 2.6% (1) |
| Ethnicity |  |
| Caucasian | 92% (36) |
| other | 5.1% (2) |
| missing | 2.6% (1) |
| BMI > 30 |  |
| yes | 23% (9) |
| no | 74% (29) |
| missing | 2.6% (1) |
| Smoking |  |
| yes | 15% (6) |
| no | 82% (32) |
| missing | 2.6% (1) |
| Institution |  |
| Graz | 26% (10) |
| Genoa | 62% (24) |
| Rennes | 13% (5) |
| Hematology oncology |  |
| yes | 10% (4) |
| no | 87% (34) |
| missing | 2.6% (1) |
| Solid organ transplant |  |
| yes | 5.1% (2) |
| no | 92% (36) |
| missing | 2.6% (1) |
| Cardiovascular disease |  |
| yes | 49% (19) |
| no | 49% (19) |
| missing | 2.6% (1) |
| Pulmonary disease |  |
| yes | 26% (10) |
| no | 72% (28) |
| missing | 2.6% (1) |
| Diabetes Mellitus |  |
| yes | 10% (4) |
| no | 87% (34) |
| missing | 2.6% (1) |
| Corticosteroids |  |
| yes | 74% (29) |
| no | 23% (9) |
| missing | 2.6% (1) |
| Tocilizumab |  |
| yes | 7.7% (3) |
| no | 90% (35) |
| missing | 2.6% (1) |
| Azithromycin |  |
| yes | 33% (13) |
| no | 62% (24) |
| missing | 5.1% (2) |
| Azole treatment at ICU admission |  |
| yes | 13% (5) |
| no | 21% (8) |
| missing | 67% (26) |
| Life Support |  |
| mechanical | 74% (29) |
| ECMO | 2.6% (1) |
| noninvasive | 5.1% (2) |
| mechanical & noninvasive | 10% (4) |
| none | 5.1% (2) |
| missing | 2.6% (1) |
| Duration ICU (days) |  |
| Mean (SD) | 31 (26) |
| valid (missing) | 38 (1) |
| Palliative (Day 28 or 32) |  |
| yes | 51% (20) |
| no | 46% (18) |
| missing | 2.6% (1) |
| Survival (at end of follow up) |  |
| yes | 33% (13) |
| no | 64% (25) |
| missing | 2.6% (1) |
| Antifungal Treatment Outcome |  |
| yes | 5.1% (2) |
| no | 5.1% (2) |
| partial | 0% (0) |
| missing | 90% (35) |
| Primary Treatment |  |
| yes | 5.1% (2) |
| no | 0% (0) |
| missing | 95% (37) |
| Antifungals initiated for CAPA |  |
| yes | 18% (7) |
| no | 0% (0) |
| missing | 82% (32) |
| Antifungal initiated |  |
| Posa Isa | 5.2% (2) |
| Isav | 0% (0) |
| IsavCasp | 2.6% (1) |
| LAmb | 0% (0) |
| Voriconazole | 10% (4) |
| missing | 82% (32) |
| BAL GM (ODI > 1) |  |
| positive | 21% (8) |
| negative | 72% (28) |
| missing | 7.7% (3) |
| BAL LFD |  |
| positive | 7.7% (3) |
| negative | 7.7% (3) |
| missing | 85% (33) |
| BAL PCR |  |
| positive | 7.7% (3) |
| negative | 49% (19) |
| missing | 44% (17) |
| BAL culture |  |
| positive | 7.7% (3) |
| negative | 90% (35) |
| missing | 2.6% (1) |
| Bronchial Aspirate culture |  |
| positive | 2.6% (1) |
| negative | 23% (9) |
| missing | 74% (29) |
| Tracheal Aspirate culture |  |
| Positive | 2.6% (1) |
| negative | 7.7% (3) |
| missing | 90% (35) |
| Tracheal Aspirate GM |  |
| positive | 2.6% (1) |
| negative | 2.6% (1) |
| missing | 95% (37) |
| Tracheal Aspirate PCR |  |
| positive | 5.1% (2) |
| negative | 21% (8) |
| missing | 74% (29) |
| Serum GM (> 0.5) |  |
| positive | 5.1% (2) |
| negative | 44% (17) |
| missing | 51% (20) |

**Table E3. Full results for significantly differentially abundant taxa determined by DESeq2 analysis when assessing CAPA diagnosis, survival, and azole treatment at ICU admission.**

| Variable | Taxa | baseMean | log2FoldChange | lfcSE | stat | p | Adjusted p |
| --- | --- | --- | --- | --- | --- | --- | --- |
| CAPA diagnosis | *Cladosporium delicatulum* | 4389.2 | 13.71 | 4.00 | 3.42 | 0.000619 | 0.003719 |
|  | *Filobasidium magnum* | 1165.8 | 12.93 | 3.13 | 4.13 | 3.59E-05 | 0.000368 |
|  | *Fungi sp* | 1017.7 | 9.18 | 2.62 | 3.51 | 0.000456 | 0.003719 |
|  | *Mycosphaerella tassiana* | 312608.4 | 20.62 | 4.01 | 5.15 | 2.65E-07 | 3.62E-06 |
| Survival (at end of follow up) | *Aspergillus fumigatus* | 8266.3 | -4.55 | 1.30 | -3.50 | 0.000462 | 0.017097 |
|  | *Candida albicans* | 83190.1 | -4.48 | 1.42 | -3.15 | 0.001611 | 0.029809 |
| Azole treatment at ICU admission | *Aspergillus fumigatus* | 943.1 | -6.33 | 2.43 | -2.6036 | 0.009225 | 0.042435 |
|  | *Candida albicans* | 66157.1 | 5.32 | 2.13 | 2.499698 | 0.01243 | 0.047648 |
|  | *Candida parapsilosis* | 8112.9 | 9.35 | 2.68 | 3.486701 | 0.000489 | 0.003749 |
|  | *Candida tropicalis* | 51931.1 | 14.52 | 2.99 | 4.85916 | 1.18E-06 | 1.36E-05 |

# Supplementary methods

**DNA extraction**

BAL DNA was extracted using a cetyltrimethylammonium bromide (CTAB) method^23^. Negative extraction controls (NECs) consisting of molecular grade Tris-EDTA (TE) buffer (Promega) were included with each sample batch. First, 500 µl samples were incubated with 0.1% dithiothreitol (Thermo Scientific) at 37 °C for 45 minutes. To lyse fungal cells, CTAB DNA Extraction Buffer (Generon Ltd) was added and a short mechanical disruption (2 x 20 seconds FastPrep-24) using glass beads (425-600µM, Sigma) was performed, followed by 3 cycles of alternated heating to 65 °C and gentle vortexing for 10 mins each. Cell lysates were treated with 4 µl RNase A (100 mg/ml, Sigma) for 30 mins prior to DNA isolation using 1:1 ratio of Phenol-Chloroform (25:24:1, Merck) and 1:1 Chloroform:isoamyl alcohol (24:1, VWR). DNA was precipitated at -20 °C overnight using 2 volumes of ice-cold 100% ethanol and 0.1 volumes of 3M sodium acetate pH 4.8. Pelleted DNA was washed in 100% ethanol and twice in 70% ethanol. Final DNA was suspended in 50 µl TE buffer.

**qPCR**

The presence of *A. fumigatus* in respiratory samples was validated using a TaqMan probe assay targeting the ITS1 region^25^. Assays were performed on an Applied Biosystems 7500 fast system using TaqMan Fast Advanced Master Mix (Thermo Fisher Scientific). Standard curves were a nine sample 10-fold dilution series beginning at 100 ng *A. fumigatus* genomes per reaction.

**PCR & Sequencing**

The ITS1 region was amplified using Nextera XT compatible versions of ITS1^24^ and ITS2degen (a degenerate version of ITS2 primer) (see Table E1). PCR setup used Phusion Green Hot Start II HF PCR master mix to create 25 µl reactions with 300 nM forward primer, 1.2 µM reverse primer and 2 µl DNA. Parameters included annealing temperature of 55 °C for 40 cycles. PCRs included positive (*Aspergillus niger* genomic DNA), negative (molecular grade water) and negative extraction controls (NECs) from DNA extractions. Controls were processed fully and sequenced alongside the BAL samples. For sequencing library preparation, samples were processed as per Illumina fungal metagenomic demonstrated protocol (Starting at ‘Clean up’ section) using Nextera XT index kit v2 (Illumina). However, this protocol was modified to include sample pooling prior to the second clean up. Libraries were sequenced 2x150 on an Illumina iSeq100, with a final library loading concentration of 50 pM.

**Data analysis**

Paired end reads were subject to quality trimming at Q30 and a minimum length filter of 75 nucleotides using bbduk^26^ (BBMap v38.22). Primer sequences were removed using Cutadapt^27^ (v1.18). Reads were mapped to UNITE database using bowtie2^28^ (v2.3.5.1). Count data was further processed in R (v4.1.3) using the following packages: phyloseq^29^ v1.38.0, vegan^30^ v2.5-7, DESeq2^31^ v1.34.0, stringr^41^ v1.4.0, ggplot2^42^ v3.3.5 and tidyr^34^ v1.2.0.

To minimise contamination in the mycobiome data, samples were subject to a multi-staged quality control process using NEC samples, along with qPCR and sequencing data. Firstly, samples from a DNA extraction batch with *A. fumigatus* detected (Ct <40) in the corresponding NEC by qPCR were excluded. Secondly, samples were excluded if they produced less than 500 fungal reads (read count filter) and/or a lower number of fungal reads than the corresponding NEC sample (NEC count filter). Of 99 initial samples, 22 were removed due to contamination of an extraction batch, 35 samples did not pass the read count filter and 3 did not pass the NEC count filter.

Manual BLAST searches confirmed the sequences assigned as *C. albicans, C. auris* and *A. fumigatus* are a 100% match to their assigned species (and *A. fumigatus* presence was also confirmed by qPCR). However, broadly, the species names described in the mycobiome data should be interpreted as candidate species designations as ITS barcoding can be unreliable for species level designations in certain cases. For example, the presence of *A. fischeri* has strong correlation with the presence of a higher abundance of *A. fumigatus* in a sample and it is likely that the *A. fischeri* sequences are a small proportion of misidentified *A. fumigatus* sequences (possibly due to sequence error introduced during the PCR stages of library preparation) as this species is very closely related to *A. fumigatus* and the ITS amplicon differs by only a single nucleotide. Abundances were standardised to the median sequencing depth. Extremely low abundance taxa were removed by only retaining those occurring > 0.2% in any sample. DESeq2 was used to identify significantly differentially abundant taxa (adjusted *p* value < 0.05 and basemean > 500). Differences in diversity (Shannon and observed OTUs) were assessed using pairwise Wilcoxon rank sum tests. PERMANOVA test was used to assess differences in Bray-Curtis ordination. A mycobiome sample was assigned as containing predominantly *A. fumigatus* if its *A. fumigatus* read counts were higher than 50% of the median sequencing depth. To determine mycobiome samples positive for high *A. fumigatus* burden by validatory qPCR, the cutoff was 0.1 haploid genome equivalents (HGE). The resulting contingency tables for sequencing and qPCR data were visualised as mosaic plots and statistical significance tested using Fisher’s exact tests.
